# Predictors and impact of non-adherence in adults with attention-deficit/hyperactivity disorder receiving OROS methylphenidate: results from a randomized, placebo-controlled trial

**DOI:** 10.1186/1471-244X-13-36

**Published:** 2013-01-24

**Authors:** J J Sandra Kooij, Michael Rösler, Alexandra Philipsen, Sandra Wächter, Joachim Dejonckheere, Annemarie van der Kolk, Michel van Agthoven, Barbara Schäuble

**Affiliations:** 1 Psycho-Medical Programs, PsyQ, Program Adult ADHD, Carel Reinierszkade 197, 2593, HR Den Haag, The Netherlands; 2 Saarland University Hospital, Neurocentre, Bdg 90.3, D-66421, Homburg/Saar, Germany; 3 Department of Psychiatry, University Hospital, Hugstetter Strasse 49, D-79095, Freiburg, Germany; 4 Janssen Medical Affairs Europe, Middle East & Africa, Johnson & Johnson Platz 5a, D-41470, Neuss, Germany; 5 SGS Life Science Services, Generaal de Wittelaan 19 A bus 5, 2800, Mechelen, Belgium; 6 Janssen–Cilag B.V, Dr. Paul Janssenweg 150, 5026 RH, Tilburg, The Netherlands; 7 Formerly Janssen Medical Affairs Europe, Middle East & Africa; now at UCB Pharma S.A, Allée de la Recherche, 60, 1070, Brussels, Belgium

**Keywords:** Adherence, Adults, ADHD, CAARS:O-SV, Efficacy, Compliance, Non-adherence, OROS methylphenidate

## Abstract

**Background:**

Medication non-adherence has an important impact on treatment efficacy and healthcare burden across a range of conditions and therapeutic areas. The aim of this analysis was to determine predictors of non-adherence and impact of non-adherence on treatment response in adults with attention-deficit/hyperactivity disorder (ADHD).

**Methods:**

Post-hoc analysis of a 13-week randomized, double-blind placebo-controlled study of OROS methylphenidate (MPH) 54 and 72 mg/day. Primary efficacy variable was the Conners’ Adult ADHD Rating Scale – Screening Version (CAARS:O-SV). Daily adherence was calculated as average daily adherence (100 × capsules taken/2), with overall adherence calculated as the average daily adherence. Predictors of adherence were assessed using mixed-effects logistic regression. Descriptive statistics were generated for change in CAARS:O-SV score for adherent (> 95% adherence) and non-adherent subjects. Predictors of change were analyzed using a mixed model.

**Results:**

Subjects were allocated to OROS MPH (54 mg, *n* = 87; 72 mg, *n* = 92) or placebo (*n* = 97). Mean adherence was 92.6% and 93.3% (OROS MPH 54 and 72 mg/day, respectively), versus 97.5% (placebo). Adherence was higher and less variable in completers. Factors significantly associated with non-adherence included female sex, shorter time since ADHD diagnosis, higher education level (completion of university) and score on the Drug Use Screening Inventory psychiatric disorders subscale. Improvements from baseline in CAARS:O-SV score were numerically greater in subjects defined as adherent than in those who were non-adherent. Significant predictors of CAARS:O-SV change in patients who completed the study included percentage adherence up to the point of assessment (*p* < 0.0001), baseline score (*p* < 0.0001) and family history of ADHD (*p* = 0.0003).

**Conclusion:**

The results of this analysis suggest that newly diagnosed patients, those with a high score on the DUSI-R psychiatric disorder scale, women, and subjects with high educational degrees may be at increased risk of non-adherence. Clinicians and policymakers should therefore pay special attention to these individuals, as non-adherence is a significant predictor of reduced response to treatment.

**Trial registration:**

EudraCT #: 2007-002111-82

## Background

Medication adherence has an important impact on treatment efficacy and healthcare burden across a range of conditions and therapeutic areas [1]. Indeed, improving adherence may have a greater impact on health than switching to a more potent or effective agent [2]. One method of improving adherence is by identifying patient and medication factors that may prevent patients from taking their medication in an optimal manner [3]. For example, adherence can be improved by simplifying medication regimens, particularly in terms of reduced frequency of dosing [1,4,5].

In a recent systematic review of 11 adherence studies in adults and children with ADHD, a very wide range was obtained for the prevalence of medication discontinuation or non-adherence (13–64%) [6]. Overall, the authors concluded that further studies are needed to investigate the extent of the problems with adherence in adults with ADHD, particularly the impact of medication non-adherence on the symptoms of ADHD and related outcomes.

Previous studies in adults with ADHD have suggested that adherence is low in this population. In a study in 66 adults receiving short-acting methylphenidate (MPH), participants reported using their medication as prescribed only half of the time on average (14.5 of 30 days) [7]. Poor adherence was best predicted by concurrent illicit substance use; younger age at MPH prescription was also associated with poor adherence, although this was not statistically significant. A study of self-reported adherence in 27 adults with ADHD who participated in a pilot study showed that mean adherence for the 2-week period before assessment was 86%, with 18% of the sample reporting less than 80% adherence, and 43% less than 90% adherence [8]. Educational level was not associated with adherence, but adherence was negatively correlated with ADHD symptoms.

According to several published guidelines, MPH is the treatment of choice for adults with ADHD [9-13]. Short-acting stimulants, however, require multiple daily intakes, which may lead to non-adherence, stigma and embarrassment with medication intake, wearing off, and possible rebound [14,15]. The osmotic release oral system (OROS) formulation was designed to deliver MPH in a controlled manner, allowing once-daily dosing in children, adolescents and adults [16,17].

While adherence to protocol in the controlled environment of a clinical trial is necessarily different from adherence to a medication regimen in real life, analysis of clinical trial data can provide useful insights into medication adherence and suggest future avenues for research and intervention. LAMDA-II was a 13-week randomized, double-blind, multicentre study in adults with ADHD randomly allocated to OROS MPH 54 or 72 mg/day, or placebo [18]. Here we report a series of post-hoc analyses from LAMDA-II that were conducted to determine factors affecting adherence with OROS MPH and the impact of non-adherence on response to treatment in terms of ADHD symptoms.

## Methods

### Study design and subjects

LAMDA-II (EudraCT #: 2007-002111-82) was a multicentre, double-blind, randomized, placebo-controlled, dose–response study conducted at 42 sites in Europe between February 2008 and April 2009. After up to 2 weeks of screening, subjects were randomly allocated to OROS MPH (54 or 72 mg/day) or matching placebo for 13 weeks. All patients received two capsules per day (1 × 18 mg + 1 × 36 mg, 2 × 36 mg or 2 × placebo). Eligible subjects were adults with a *Diagnostic and Statistical Manual of Mental Disorders* (4th ed., Text Revision, *DSM-IV-TR*) diagnosis of ADHD, confirmed using Conners’ Adult ADHD Diagnostic Interview Part II (CAADID) for *DSM-IV*. To be eligible for the study, subjects were required to have a score of ≥ 24 on the observer-rated Conners’ Adult ADHD Rating Scale—Screening Version (CAARS:O-SV). The Structured Clinical Interview for *DSM-IV* Axis I disorders (SCID-I) was used to evaluate the presence of co-morbidities and to exclude other disorders. The study conforms to the requirements of the 1964 Declaration of Helsinki. The research protocol was approved by the ethics committees at each site (Table 1), and all participants gave written informed consent. Full details of the study design and eligibility criteria have been published previously [18].

**Table 1:** Ethics committees

**Country**	**Committee**
**Norway**	Regional komité for medisinsk og helsefaglig forskningsetikk, Sør-Øst-Norge (REK Sør-Øst)
**France**	Comité de Protection des Personnes – Ile de France VI
**Sweden**	Regionala etikprövningsnämnden i Stockholm
**Denmark**	Den Videnskabsetiske Komité for Region Midtjylland
**Netherlands**	UMC St. Radboud
	Adviescommissie Mensgebonden
	METIGG (Kamer Noord)
**Spain**	Secretaría del CEIC, Servicio Farmacología Clínica
	Hospital Clínico San Carlos
	Secretario del CEIC, Servicio de Farmacia, Hospital Vall d’Hebrón
	Hospital Gregorio Marañón, Oficina Técnica CEIC-A1
**Belgium**	Ethische Commissie, Sint-Vincentiusziekenhuis vzw.
	vzw Emmaüs Ethisch Comité
	Comité Ethique, Hôpital Psychiatrique Le Chêne aux Haies
	Faculteit Geneeskunde, Commissie Medische Ethiek/Klinischonderzoek
**UK**	East London & The City Research, Ethics Committee 1
	Cambridgeshire 2 Research Ethics Committee
	South West Wales REC
**Finland**	Pohjois-Pohjanmaan sairaanhoitopiirin eettinen toimikunta, Hallintokeskus
**Germany**	Ethik-Kommission des Landes Berlin
	Ethik-Kommission der Ärztekammer, Schleswig-Holstein
	Ethik-Kommission der Medizinischen, Fakultät der Universität Duisburg-Essen, Universitätsklinikum
	Ethik-Kommission der Bayerischen Landesärztekammer
	Ethik-Kommission bei der Ärztekammer des Saarlandes
	Ethik-Kommission der Albrecht-Ludwigs-Universität Freiburg
	Ethikkommission der Medizinischen, Fakultät der LMU München
	Medizinische Ethik-Kommission II der Fakultät für Klinische Medizin, Mannheim der Ruprecht-Karls-Universität Heidelberg
	Ethikkommission der Medizinischen Fakultät der Bayerischen Julius-Maximilians-Universität Würzburg
	Ethikkommission der Medizinischen Fakultät der Heinrich-Heine-Universität
**Switzerland**	Etikkommission bei der Basel EKBB

### Assessments

The primary efficacy variable in the study was the investigator-rated CAARS:O-SV, which was assessed after 1 week and then every 2 weeks thereafter. The CAARS:O-SV comprises 18 items corresponding to the 18 *DSM-IV*–defined ADHD symptoms and provides a total score and two subscale scores (Inattention and Hyperactivity/Impulsivity), with higher scores indicating greater ADHD symptoms [19]. The CAARS:O-SV has shown very high model fit in healthy control and ADHD patient samples, and the reliability and cross-cultural validity of the instrument have been demonstrated [20]. The presence of substance use disorders was assessed using the Drug Use Screening Inventory Revised (DUSI-R), a 159-item self-administered instrument [21]. The 159 questions are subdivided into 11 domains: drug and alcohol use frequency, substance use, behavior pattern, health status, psychiatric disorder, social competence, family system, school performance, work adjustment, peer relationship and leisure/recreation.

Daily adherence to therapy was calculated as the ratio of capsules taken on that day to capsules prescribed by the protocol (i.e. 100 × number of capsules taken on that day/2). Overall adherence was calculated as the average daily adherence during the 13-week double-blind study. Patients were defined as adherent if their overall adherence was > 95%.

### Statistical analysis

For the purposes of this post-hoc analysis, the main analysis set was defined as all randomized subjects who received at least one dose of study medication and had at least one post-baseline efficacy assessment. Because adherence is strongly related to study discontinuation, we replicated our analyses on a ‘completers’ subgroup, which was defined as all subjects in the main analysis set who completed all CAARS:O-SV assessments as scheduled in the study protocol. This analysis served as a sensitivity check for the analysis on the main analysis set, as any findings from the analyses on the completers cannot be attributed to (early) discontinuation.

Predictors of daily adherence were assessed using a mixed-effects logistic regression model. This model is appropriate for the analysis of a binary variable (adherent/non-adherent), while it also incorporates the longitudinal design (one outcome per day per patient) as a hierarchical structure in which daily adherence is nested within patient [22]. In addition, this model allows for the inclusion of time-varying covariates. The following variables were included in the model: age; sex; history of mood or anxiety disorder, country; treatment group; adult ADHD subtype (predominantly inattentive, predominantly hyperactive–impulsive or combined); age at ADHD diagnosis; time since ADHD diagnosis; family history of ADHD; highest level of education completed; employment status; prior use of MPH; concomitant treatment for anxiety disorder; concomitant treatment for depression; baseline CAARS:O-SV total score; DUSI subscale scores; and day of the week.

To explore the impact of adherence on efficacy, descriptive statistics were generated for change in CAARS:O-SV score from baseline for adherent (> 95% overall adherence) and non-adherent subjects. In addition, a statistical analysis on potential predictors of change in CAARS:O-SV score on all time points per patient during treatment was performed using a mixed model. The following independent variables were considered: prior adherence (i.e., percentage adherence in the period before each CAARS:O-SV assessment); day of the week and season as time-varying covariates, age, sex, country, treatment group, ADHD subtype, time since ADHD diagnosis, family history of ADHD (yes or no), highest level of education completed and employment status (employed vs. unemployed), baseline CAARS score, history of mood and anxiety disorder (yes or no), prior use of MPH (yes or no), age at ADHD diagnosis, all DUSI scales, concomitant treatment for anxiety disorder (yes or no) and concomitant treatment for depression (yes or no), as independent variables.

The following testing strategy was followed for both statistical models. All independent variables were first tested for univariately, and included in the multivariate model in case of *p* < 0.20. Interaction of each independent variable with treatment group was tested in the multivariate model and kept in the model if *p* < 0.1. Independent variables with *p* > 0.1 were removed from the multivariate model in a backwise fashion, and variables with the largest *p*-value were removed first. Only the final model is presented in this manuscript. Restricted likelihood distance and Cook’s *d* were used to detect influential observations. No influential observations were dropped.

### Ethics committees

## Results

### Subjects and disposition

The main analysis set consisted of 276 subjects allocated to OROS MPH 54 mg (*n* = 87) or 72 mg (*n* = 92), or to placebo (*n* = 97). Baseline characteristics are shown in Table 2. The completer population included 67 subjects (69.1%) in the placebo arm, 55 subjects (63.2%) in the 54-mg arm and 54 subjects (58.6%) in the 72-mg arm. Reasons for discontinuation included lack of efficacy (*n* = 14; 14%) in the placebo arm, and adverse events in the OROS MPH 54-mg (*n* = 15; 17%) and 72-mg (*n* = 19; 21%) arms (Table 3). Full details of the study population have been published previously [5].

**Table 2:** Baseline characteristics and disease history

**Characteristic**	**Placebo**	**OROS MPH**	**OROS MPH**
	**(*n = *97)**	**54 mg (*n *= 87)**	**72 mg (*n = *92)**
Age, years			
Mean ± SD	35.5 ± 8.8	35.5 ± 11.8	35.8 ± 10.1
Median (range)	36 (18-57)	33 (18-64)	35 (18-60)
Sex, *n* (%)			
Female	45 (46.4)	44 (50.6)	42 (45.7)
Male	52 (53.6)	43 (49.4)	50 (54.3)
Educational status, *n* (%)			
Primary school	7 (7.2)	8 (9.2)	8 (8.7)
Secondary school	40 (41.2)	24 (27.6)	29 (31.5)
High school	29 (29.9)	29 (33.3)	26 (28.3)
University	21 (21.6)	26 (29.9)	29 (31.5)
Age at ADHD diagnosis, years (mean ± SD)	31.9 ± 12.8	31.2 ± 15.1	32.4 ± 13.0
Adult ADHD type, *n* (%)			
Combined	73 (75.3)	59 (67.8)	62 (67.4)
Predominantly inattentive	23 (23.7)	26 (29.9)	28 (30.4)
Predominantly hyperactive– impulsive	1 (1.0)	2 (2.3)	2 (2.2)
Family history of ADHD, *n* (%)	53 (54.6)	55 (63.2)	52 (56.5)
History of mood and anxiety disorder, *n* (%)	50 (51.5)	39 (44.8)	39 (42.4)
Baseline CAARS-O:SV (mean ± SD)	36.5 ± 6.1	35.7 ± 6.8	37.3 ± 6.4

**Table 3:** Reasons for discontinuation during the study

**Reason for discontinuation, *n *(%)**	**Placebo**	**OROS MPH**	
	**(*n *= 97)**	**54 mg/day**	**72 mg/day**
		**(*n *= 87)**	**(*n *= 92)**
Discontinued	29 (29.9)	32 (36.8)	37 (40.2)
Adverse event	2 (2.1)	15 (17.2)	19 (20.7)
Lack of efficacy	14 (14.4)	1 (1.1)	4 (4.3)
Noncompliance	3 (3.1)	5 (5.7)	5 (5.4)
Consent withdrawal	4 (4.1)	2 (2.3)	3 (3.3)
Loss to follow-up	5 (5.2)	1 (1.1)	0
Sponsor’s decision	0	2 (2.3)	0
Ineligibility to continue the study	0	1 (1.1)	1 (1.1)
Other	1 (1.0)	5 (5.7)	5 (5.4)

### Adherence

**Figure 1:**
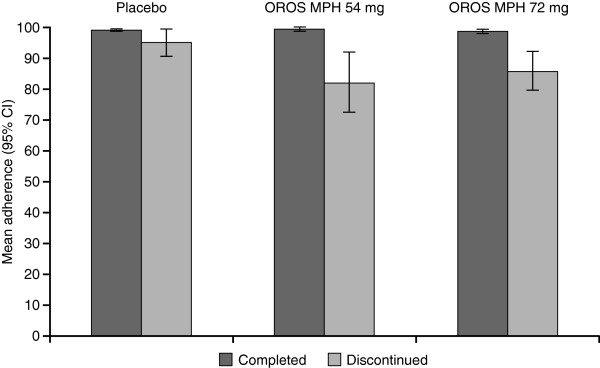
Mean adherence in patients who completed the study and in those who discontinued.

Mean (± SD) adherence was 92.6% ± 17.6 and 93.3% ± 13.4 in the 54- and 72-mg/day OROS MPH groups, respectively, versus 97.5% ± 6.8 in the placebo group (main analysis set). Mean adherence was higher and less variable in the completers subgroup compared with those who discontinued, especially in those receiving active treatment (Figure 1).

**Figure 2:**
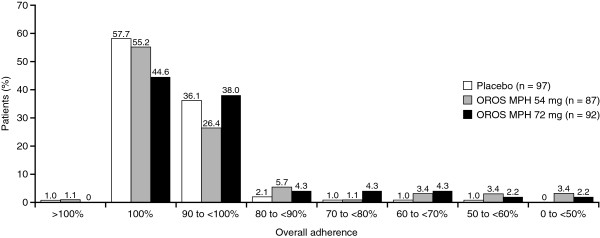
Overall adherence.

Overall adherence with study medication intake of 100% was observed in 55.2% (*n* = 48), 44.6% (*n* = 41) and 57.7% (*n* = 56) of subjects receiving OROS MPH 54 or 72 mg, or placebo, respectively (one subject in each of the placebo and OROS MPH 54-mg arms reported taking four capsules per day on one day and had no record of missed doses, and therefore had an adherence > 100%) (Figure 2). No systematic trend in adherence could be found.

### Predictors of adherence

In the mixed-effects logistic regression model on daily adherence, several significant predictors of adherence were identified (Table 4). Overall, men tended to be more adherent than women (*p* = 0.0892; main analysis set), and subjects receiving placebo were more adherent than those receiving OROS MPH (*p* = 0.0221 and *p* = 0.0371 for the 54-mg and 72-mg doses, respectively). Adherence increased with increasing time since diagnosis of ADHD (*p* = 0.007), and subjects who completed university were significantly less likely to be adherent than those who completed high school (*p* = 0.0284). The higher (i.e. more problematic) the score on the DUSI psychiatric disorder scale at baseline, the less likely subjects were to be adherent (*p* = 0.0252). In the completer analysis, there was no significant difference in adherence between OROS MPH groups and placebo (Table 4). In addition, male sex, DUSI psychiatric disorder score and time since ADHD diagnosis were no longer significantly related to adherence (and therefore do not appear in the final model, as shown in Table 4), but DUSI health status score at baseline was. As in the overall analysis, subjects who completed university were significantly less likely to be adherent than those who completed their education after high school (*p* < 0.0001) or primary school (*p* = 0.0197).

**Table 4:** Predictors of adherence

**Variable**	**Main analysis set**		**Completer population**	
	**Point estimate**	***p*-value**	**Point estimate**	***p*-value**
Treatment: OROS MPH 54 mg	−1.0472	0.0221	−0.0645	0.7712
Treatment: OROS MPH 72 mg	−0.9122	0.0371	−0.2533	0.2244
Sex (Male)	0.6374	0.0892	–	–
Country	–^a^	NS	–^b^	–^b^
DUSI: psychiatric disorder	−0.0296	0.0252	–	–
DUSI health status	−0.0238	0.0563	−0.0182	0.0012
Education level: primary	0.1759	0.8102	0.9829	0.0107
Education level: secondary	0.4870	0.2963	0.1399	0.5012
Education level: high school	1.0683	0.0284	1.2055	< 0.0001
Time since ADHD diagnosis	0.0770	0.0070	–	–

^a^ Point estimates for individual countries ranged from −0.4944 to +2.6307; none were statistically significant.

^b^ Point estimates for individual countries ranged from −2.1366 to +3.2225; values were statistically significant for France (point estimate: −2.1366 [*p* < 0.0001]) and Sweden (estimate: −1.4175 [*p* = 0.0074]).

### Impact of non-adherence on efficacy

**Figure 3:**
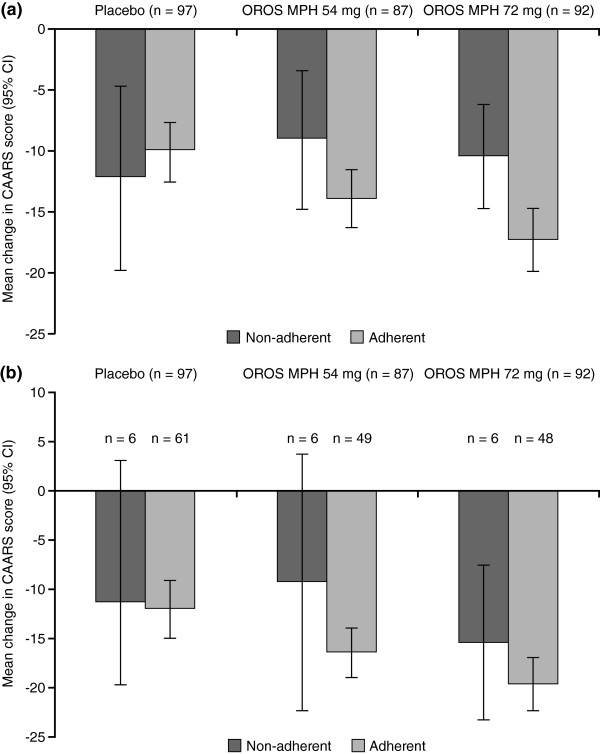
**Mean (95% CI) change from baseline to endpoint in CAARS:O-SV score.** Subjects defined as adherent (> 95% overall adherence) or non-adherent in the (**a**) main analysis set; and (**b**) completer population.

For subjects receiving OROS MPH, decreases (improvements) from baseline in CAARS:O-SV score were numerically greater in subjects defined as adherent than in those who were not, in both the main analysis set and completer population (Figure 3).

**Figure 4:**
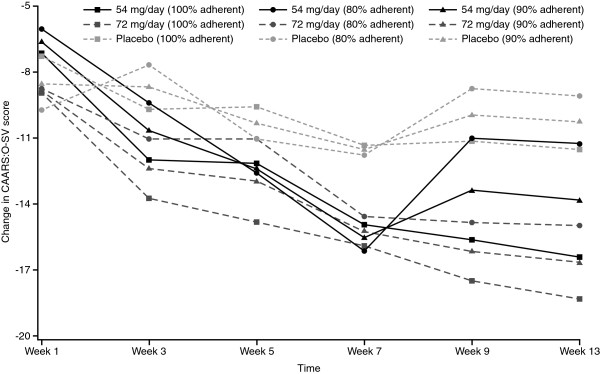
Estimated change in CAARS:O-SV score over time by treatment group for given levels of adherence.

In the main analysis set, significant predictors of change in CAARS:O-SV score (Table 5) included baseline CAARS:O-SV score (*p* < 0.0001), study time point (*p* < 0.0001) and percentage overall adherence in the study before the CAARS:O-SV assessment (*p* < 0.0001). The decrease in CAARS:O-SV was significantly larger for both MPH groups versus placebo. The greatest improvement in CAARS:O-SV score occurred early in the study, and the decrease in CAARS:O-SV over time differed between the treatment groups, although the level of adherence, particularly in the 72-mg arm, affected change in CAARS:O-SV throughout the study (Figure 4). Finally, subjects with a family history of ADHD showed greater improvement (*p* = 0.0104), and the longer the time since diagnosis of ADHD, the less the degree of improvement (*p =* 0.0072).

**Table 5:** Regression analysis of variables affecting change in CAARS:O-SV score (main analysis set)

	**Estimate**	***p*-value**	**Difference in least squares means**	***p*-value**
Baseline	–0.4203	0.0007	–	–
Prior adherence	–0.0145	0.7530	–	–
Treatment group:				
54 mg	2.6840	0.7436	–2.7528	0.0145
72 mg	12.2774	0.1441	–4.5799	< 0.0001
Baseline × Treatment group:				
54 mg	0.1473	0.3922	–	–
72 mg	–0.2533	0.1448	–	–
Time point (versus Week 13)				
Week 1	3.9770	0.0003	7.1825	0.0001
Week 3	1.8149	0.0767	3.3609	< 0.0001
Week 5	1.5768	0.0923	2.8016	< 0.0001
Week 7	–0.0884	0.9186	0.8591	0.1023
Week 9	0.3289	0.6653	0.5197	0.2625
Time point × Treatment group				
Week 1 × 54 mg	4.6804	0.0037	0.3118	0.8088
Week 1 × 72 mg	4.8763	0.0022	–1.6352	0.1917
Week 3 × 54 mg	2.1269	0.1603	–2.2417	0.0908
Week 3 × 72 mg	2.5112	0.0914	–4.0003	0.0020
Week 5 × 54 mg	1.8791	0.1753	–2.4895	0.0667
Week 5 × 72 mg	1.7954	0.1878	–4.7161	0.0004
Week 7 × 54 mg	0.7517	0.5571	–3.6169	0.0101
Week 7 × 72 mg	2.0907	0.0980	–4.4208	0.0013
Week 9 × 54 mg	0.2565	0.8192	–4.1121	0.0045
Week 9 × 72 mg	0.3160	0.7777	–6.1955	< 0.0001
Treatment group × adherence				
54 mg	–0.1276	0.0197	–	–
72 mg	–0.0976	0.0721	–	–
Female sex	–1.5429	0.0949	–	–
No family history of ADHD	2.4018	0.0104	–	–
Time since ADHD diagnosis	0.1403	0.0072	–	–

In the completer population, higher baseline CAARS:O-SV score (*p* < 0.0001), higher percentage prior adherence (*p* < 0.0001), longer time in study (*p* < 0.01 for 72-mg arm from Week 5 onwards and *p* < 0.05 for 54-mg arm from Week 7 onwards) and family history of ADHD (*p* = 0.0003) were significant predictors of change in CAARS:O-SV score (Table 6). Again, both MPH groups showed a significantly larger improvement compared with placebo, and the decrease in CAARS:O-SV score over time was different between the treatment groups.

**Table 6:** Regression analysis of variables affecting change in CAARS:O-SV score (completer population)

**Variable**	**Point estimate**	***p*-value**	**Difference in least squares means**	***p*-value**
Treatment group				
54 mg	–4.6356	0.0039	–3.0139	0.0247
72 mg	–6.9787	< 0.0001	–4.6818	0.0004
Baseline CAARS:O-SV score	–0.3721	< 0.0001	–	–
Prior adherence	–0.1423	< 0.0001	–	–
No family history of ADHD	3.9342	0.0003	–	–
Time point × Treatment group				
Week 1 × 54 mg	4.6305	0.0078	–0.0051	0.9975
Week 1 × 72 mg	6.0762	0.0004	–0.9025	0.5672
Week 3 × 54 mg	2.3155	0.1476	–2.3201	0.1468
Week 3 × 72 mg	3.2686	0.0390	–3.7101	0.0189
Week 5 × 54 mg	2.1577	0.1294	–2.4779	0.1213
Week 5 × 72 mg	2.2344	0.1121	–4.7443	0.0027
Week 7 × 54 mg	0.6507	0.6197	–3.9849	0.0128
Week 7 × 72 mg	1.9795	0.1268	–4.9992	0.0016
Week 9 × 54 mg	–0.0242	0.9829	–4.6598	0.0036
Week 9 × 72 mg	0.2229	0.8419	–6.7558	< 0.0001
DUSI: family system	–0.0551	0.0583	–	–

## Discussion

In the present study, adherence to OROS MPH treatment was generally high, as might be expected in a randomized, placebo-controlled trial. Self-reported adherence in real-life settings is, however, substantially lower [7,8]. Factors associated with reduced adherence in the present study included score on the DUSI-R psychiatric disorder scale, completing university versus high school, and shorter time since diagnosis of ADHD; and men were more adherent than women. Adherence was also higher in subjects receiving placebo than in those receiving OROS MPH. In particular, the discontinuers in the MPH groups had a low adherence. In the patients who completed the trial, there was no difference in adherence between the treatment groups. In previous studies that looked at factors associated with adherence in adults with ADHD, adherence was significantly correlated with ADHD symptoms, substance abuse and younger age [7,8]. These studies had small sample sizes, however, and were based on patient reports of adherence, so direct comparison with the present study is difficult.

It is possible that some of the factors identified as contributing to non-adherence (e.g. education, time since diagnosis) may, in fact, reflect some degree of misdiagnosis of ADHD, with subsequent impact on adherence and study discontinuation. For example, the disorder is highly genetic, is lifelong, and is likely to interfere with completion of education. Furthermore, some of its symptoms may be mimicked by other psychiatric disorders (e.g. anxiety or depression may produce difficulty concentrating or maintaining focus on current activities). It is important to note, however, that a short time since diagnosis is common in adults with ADHD, as they are unlikely to have been diagnosed during childhood in Europe. The impact of short time since diagnosis on adherence may therefore reflect lower acceptance of the disorder and less motivation for treatment. Similarly, lack of family history could suggest a subgroup who have ADHD that is less heritable, or where the patient does not know or realise that ADHD is present in the family (not uncommon when one is not yet familiar with the clinical presentation of ADHD). Psychiatric comorbidity in patients with ADHD may lead to low adherence through impact of MPH on other symptoms, such as anxiety or depression, while substance abuse is clearly associated with non-attendance, stagnation of treatment and interference with evaluation of medication. Similarly, completion of higher education does not necessarily point to an incorrect diagnosis; more intelligent individuals with ADHD tend to achieve higher levels of education than those of lower intelligence, yet still experience impairments relative to their potential.

The analysis of change in CAARS:O-SV showed that lower adherence was significantly associated with less improvement in ADHD symptoms, particularly in subjects receiving OROS MPH. The other notable predictor of treatment efficacy was the presence of a family history of ADHD; this may be because subjects with affected family members may have had access to greater support. As expected, baseline CAARS:O-SV score was also significantly associated with improvement in CAARS:O-SV, as was time since ADHD diagnosis. As non-adherence is related to study discontinuation, the analysis was repeated on study completers and it was again shown that non-adherence significantly affected the level of improvement in CAARS:O-SV. There were, however differences between the main analysis set and the completer population, which may be related to the impact of discontinuation in the main analysis set, and may also have been affected by the decreased statistical power in the smaller population or the lower variability in adherence in the completer population.

A main strength of the present study is the size of the cohort, although it should be noted that this was a post-hoc analysis and the study was not designed to assess adherence. In addition, identification of factors that are statistically associated with adherence does not always translate into clinical relevance [23]. A randomized, controlled trial may not be the best setting in which to evaluate adherence, which may be higher than in the ‘real world,’ as suggested by previous small studies in adults with ADHD [7,8]. The generalizability of the study findings may therefore be limited to clinical trials in which patient inclusion is strictly controlled, excluding patients with, for example, psychiatric co-morbidity. It should also be noted that there are numerous possible methods of assessing adherence, including patient reports, carer reports, pill counts, pharmacy data, electronic measurement and measurement of plasma drug levels; but none of these methods is ideal. In the present study, it is possible that measurements of adherence were affected by patients deliberately returning fewer capsules to give the impression that their adherence was higher [24]. Furthermore, one important predictor of adherence—number of medication doses—could not be analysed in the present study, as all patients received a single daily dose of OROS MPH or placebo. Finally, the potential impact of selecting a cut-off for the definition of ‘adherent’ other than 95% was not investigated.

Concepts of adherence are continuing to evolve, and the factors involved in adherence can be extended to a broader concept of ‘concordance,’ as recently introduced by David Coghill (J. J. S. Kooij; personal communication). This describes something beyond adherence, namely mutual agreement between patient and physician with the need for medication and the correct way to take it. Patient education is a key part of achieving concordance.

## Conclusions

The results of this study suggest that women, newly diagnosed patients, those with a high score on the DUSI-R psychiatric disorder scale, and subjects with high educational degrees, at least in the context of this study, seem to have a higher risk of non-adherence. In this regard, routine assessment of these possible predictors—as well as patients’ attitudes towards medication, systematic evaluation of target symptoms and side effects, and adherence by patient self-report and follow-up with patients at risk of poor adherence—may be valuable [23]. Furthermore, physicians and other healthcare workers should encourage good adherence by education of, and discussion with, the patient about all aspects involved in treatment, to maximize treatment efficacy. While adherence with OROS MPH in this study was generally high, non-adherence was associated with a reduced response to treatment, as measured by the CAARS:O-SV.

## Competing interests

JJSK has been a speaker for Janssen–Cilag BV, Shire and Eli Lilly BV, and has received unrestricted research grants from Janssen–Cilag BV and Shire. MR is a consultant for Janssen–Cilag, Medice, Lilly and Shire, and is a member of the speaker’s bureau of Janssen–Cilag, Medice and Shire. AP is a consultant for Janssen–Cilag, Lilly, Medice, and Shire, and is a member of the speaker’s bureau of Janssen–Cilag, Medice, Shire, and Novartis. SW and MvA are employees of Janssen–Cilag EMEA. JD is a consultant working on behalf of SGS–Life Science Services, a company employed by Janssen–Cilag EMEA to provide statistical analysis. AvdK is an employee of Janssen–Cilag B.V. BS was an employee of Janssen–Cilag EMEA at the time the manuscript was developed.

## Authors’ contributions

JJSK was an investigator in the LAMDA-II trial, and was involved in the interpretation of the data, and the drafting and reviewing of the manuscript. MR was an investigator in the LAMDA-II trial, and was involved in the interpretation of the data, and the drafting and reviewing of the manuscript. AP was an investigator in the LAMDA-II trial, and was involved in the interpretation of the data, and the drafting and reviewing of the manuscript. SW was involved in the interpretation of the data, and the drafting and reviewing of the manuscript. JD carried out the statistical analyses and was involved in the interpretation of the data, and the drafting and reviewing of the manuscript. AvdK was involved in the interpretation of the data, and the drafting and reviewing of the manuscript. MvA was involved in the interpretation of the data, and the drafting and reviewing of the manuscript. BS was involved in the interpretation of the data, and the drafting and reviewing of the manuscript. All authors read and approved the final manuscript.

## Pre-publication history

The pre-publication history for this paper can be accessed here:

http://www.biomedcentral.com/1471-244X/13/36/prepub
